# IUC26700-80 Outcomes of dual immunotherapy versus other first line treatments for metastatic renal carcinoma

**DOI:** 10.1093/oncolo/oyag286.029

**Published:** 2026-08-21

**Authors:** E Woods, S Growcott, A Gee

**Affiliations:** Royal United Hospital, Bath - United Kingdom; Royal United Hospital, Bath - United Kingdom; Royal United Hospital, Bath - United Kingdom

## Abstract

**Background:**

The Checkmate trial demonstrated that patients with previously untreated advanced renal cell carcinoma who received first-line dual immunotherapy with nivolumab plus ipilimumab showed significantly higher objective response rates than those treated with sunitinib monotherapy. In light of these findings, this study aimed to compare the real-world outcomes of patients with metastatic renal cell carcinoma treated at the Royal United Hospital, Bath, UK, who received first-line dual immunotherapy (IO) with those treated using alternative first-line therapeutic options for advanced disease, including tyrosine kinase inhibitors (TKIs) or combination IO plus TKI therapy.

**Methods:**

This is a single-centre, retrospective cohort study looking at data from 2018 to 2026. There were 112 patients included (IO-IO n  =  23, TKI n  =  68, IO-TKI n  =  21, combined = 89). The primary outcome was duration of treatment. Secondary outcomes included objective response rates, complete response rate, incidence of treatment-related adverse events and reasons for treatment discontinuation.

**Results:**

The median duration of treatment was 7 months for those treated with IO-IO, and 11 months for those treated with either TKI or IO-TKI. The objective response rate was 52% versus 71% (p  =  0.09), and the complete response rate was 9% versus 3%, respectively. 100% of patients receiving IO-IO were recorded as having treatment-related adverse events compared to 97% of patients receiving TKI or TKI plus IO. Treatment-related adverse events led to discontinuation of treatment in 13% of patients receiving IO-IO and 11% of those receiving TKI or IO-TKI. Study limitations included the relatively small number of patients and its retrospective design, which relied on individual clinicians’ documentation of IO toxicities and grades.

**Conclusions:**

The complete response rate was higher in patients receiving IO-IO. The overall objective response rate and treatment duration were lower for patients receiving nivolumab plus ipilimumab compared to other first-line treatment options; however, they were similar to those demonstrated in the Checkpoint trial^1^. The incidence of treatment-related adverse events was also consistent with the checkpoint trial^1^. Future studies could also include patient-reported outcomes of any treatment toxicities and the impact of these on their quality of life. This would enable more informed shared decision-making.

